# Induction of Apoptosis by 11-Dehydrosinulariolide via Mitochondrial Dysregulation and ER Stress Pathways in Human Melanoma Cells 

**DOI:** 10.3390/md10081883

**Published:** 2012-08-22

**Authors:** Tzu-Rong Su, Feng-Jen Tsai, Jen-Jie Lin, Han Hsiang Huang, Chien-Chih Chiu, Jui-Hsin Su, Ya-Ting Yang, Jeff Yi-Fu Chen, Bing-Sang Wong, Yu-Jen Wu

**Affiliations:** 1 Antai Medical Care Cooperation Antai Tian-Sheng Memorial Hospital, Pingtung 92842, Taiwan; Email: a081002@mail.tsmh.org.tw (T.-R.S.); a098123@mail.tsmh.org.tw (B.-S.W.); 2 Department of Beauty Science, Meiho University, Pingtung 91202, Taiwan; Email: x00002036@meiho.edu.tw (F.-J.T.); hhuang.adsl@msa.hinet.net (H.H.H.); 3 Graduate Institute of Veterinary Medicine, National Pingtung University of Science and Technology, Pingtung 91202, Taiwan; Email: q87634@yahoo.com.tw; 4 Department of Biotechnology, Kaohsiung Medical University, Kaohsiung 80761, Taiwan; Email: woodnettle2002@gmail.com (C.-C.C.); yifuc@kmu.edu.tw (J.Y.-F.C.); 5 National Museum of Marine Biology and Aquarium, Pingtung 94446, Taiwan; Email: x2219@nmmba.gov.tw; 6 Chemistry Department, National Sun Yat-Sen University, No. 70, Lienhai Rd., Kaohsiung 80424, Taiwan; Email: tinayang101@gmail.com

**Keywords:** melanoma, 11-dehydrosinulariolide, mitochondrial dysregulation, ER stress

## Abstract

In this study the isolated compound 11-dehydrosinulariolide from soft coral Sinularia *leptoclados* possessed anti-proliferative, anti-migratory and apoptosis-inducing activities against A2058 melanoma cells. Anti-tumor effects of 11-dehydrosinulariolide were determined by MTT assay, cell migration assay and flow cytometry. Growth and migration of melanoma cells were dose-dependently inhibited by 2–8 μg/mL 11-dehydrosinulariolide. Flow cytometric data indicated that 11-dehydrosinulariolide induces both early and late apoptosis in melanoma cells. It was found that the apoptosis induced by 11-dehydrosinulariolide is relevant to mitochondrial-mediated apoptosis via caspase-dependent pathways, elucidated by loss of mitochondrial membrane potential (∆Ψm), release of cytochrome *C*, activation of caspase-3/-9 and Bax as well as suppression of Bcl-2/Bcl-xL. The cleavage of PARP-1 suggested partial involvement of caspase-independent pathways. Immunoblotting data displayed up-regulations of PERK/eIF2α/ATF4/CHOP and ATF6/CHOP coupling with elevation of ER stress chaperones GRP78, GRP94, calnexin, calreticulin and PDI, implicating the involvement of these factors in ER stress-mediated apoptosis induced by 11-dehydrosinulariolide. The abolishment of apoptotic events after pre-treatment with salubrinal indicated that ER stress-mediated apoptosis is also induced by 11-dehydrosinulariolide against melanoma cells. The data in this study suggest that 11-dehydrosinulariolide potentially induces apoptosis against melanoma cells via mitochondrial dysregulation and ER stress pathways.

## 1. Introduction

Melanoma is a malignant tumor of cutaneous melanocytes. Incidence of melanoma is usually lower than other skin cancers such as basal cell cancer and squamous cell cancer. However, melanoma is more invasive and has a higher lethality than other skin cancers [1]. Melanoma incidence has been estimated to reach 70,230 cases in 2011 in the US [2]. In the regions with sunny climates like Australia, New Zealand, North America, Latin America, and northern Europe melanoma occurs the most [3]. The metastases of melanoma are clinically crucial in diagnosed patients since most systemic treatments for metastatic melanoma exert non-obvious effects [4,5]. Surgery is still the most effective measure for malignant melanoma patients with small and thin neoplastic lesions [6]. The survival rate is dependent on the diagnostic stage of melanoma [7]. Other medical treatments such as chemotherapy (with dacarbazine) and immunotherapy (with interleukin-2 or interferon) have also been investigated [8,9,10]. However, their achievements clinically applied in metastatic melanoma are still limited [11]. Therefore, it is necessary to investigate and discover new effective drugs and therapies against human malignant melanoma. 

The induction of cell death by apoptosis critically benefits cancer therapy development [12,13]. Apoptotic processes can be triggered either via extrinsic pathways or intrinsic pathways [14,15]. Recent studies have implied that intrinsic pathways are initiated by association with stress in the mitochondria or endoplasmic reticulum (ER) [16]. Processes of mitochondrial dysregulation have been shown as major events during apoptosis while Bcl-2 family like Bax and Bak as well as alteration of mitochondrial membrane potential result in the release of mitochondrial apoptotic factors [17]. Cytochrome *C* release from mitochondrial inter-membrane spaces initiates subsequent activation of caspase-9 as the downstream effector caspase-3 is further activated [17,18]. ER is responsible for synthesis of cellular regulatory proteins, protein folding and intracellular calcium homeostasis [19]. The abnormality of protein folding or calcium imbalance in ER is able to trigger ER stress and this subsequently induces self-rescuing or destruction responses in cells [20].

Some chemical compounds isolated from soft coral in a recent study were found to possess cytotoxic activities in tumor cells [21,22,23,24]. These compounds derived from marine products were shown to exert apoptosis-induced effects against human hepatocellular carcinoma [25], hormone-resistant prostate cancer cells [26], oral squamous cell carcinoma [27] and bladder cancer carcinoma [23]. Herein we examined the anti-tumor and apoptosis-inducing effects of 11-dehydrosinulariolide on melanoma cells using MTT assay, cell migration assay and flow cytometric analysis. The potential pathways of apoptosis induced by 11-dehydrosinulariolide in melanoma cells were determined by mitochondrial membrane potential measurement, apoptotic inhibition test and immunoblotting. We revealed crucial data on the cytotoxic activities and several apoptotic pathways of 11-dehydrosinulariolide in melanoma cells *in vitro*. 

## 2. Results

### 2.1. The Anti-Tumor Effects of 11-Dehydrosinulariolide on A2058 Cells

**Figure 1:**
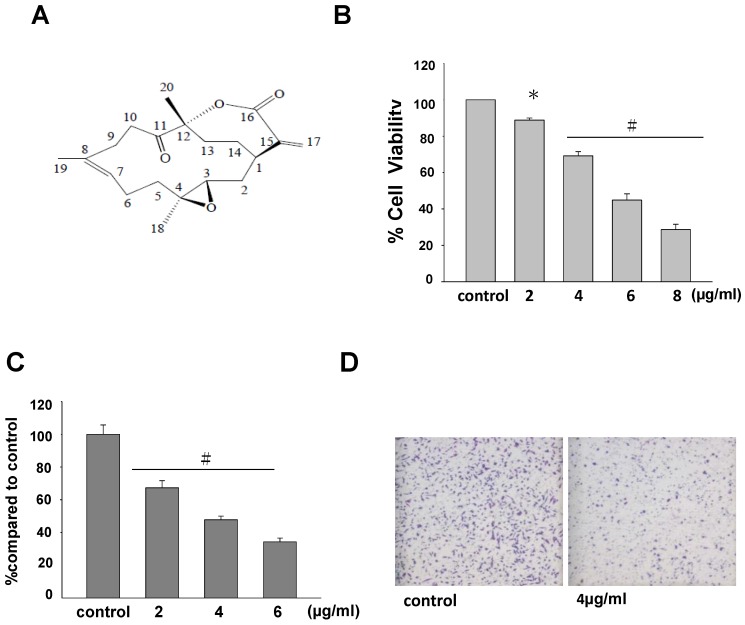
Anti-proliferative and anti-migratory effects of 11-dehydrosinulariolide at different concentrations against A2058 melanoma cells (**A**) The chemical structure of 11-dehydrosinulariolide; (**B**) The viability of A2058 melanoma cells was dose-dependently inhibited by treatment with 2–8 μg/mL 11-dehydrosinulariolide for 24 h, represented by MTT assay (* *P* < 0.05; # *P* < 0.001); (**C**) Cell migration assay showed that 11-dehydrosinulariolide from 2 μg/mL to 6 μg/mL dose-dependently suppresses A2058 cell migration (# *P* < 0.001) and (**D**) Migrated A2058 cells were clearly reduced (4 μg/mL 11-dehydrosinulariolide treated) compared with control at 100× magnification.

The data of MTT assay showed that proliferation of A2058 melanoma cells was dose-dependently inhibited by 2 to 8 μg/mL of 11-dehydrosinulariolide. When the exposure to 11-dehydrosinulariolide reached 6 μg/mL, melanoma, cell viability was reduced to the level approximately 48% of the control (Figure 1). The half maximal inhibitory concentration (IC50) of 11-dehydrosinulariolide for MTT assay is approximately 5.8 μg/mL. Cell migration data displayed that 2–6 μg/mL 11-dehydrosinulariolide suppressed melanoma cell migration in a dose-dependent manner as the suppression rates were approximately 32%, 51% and 73% for 2, 4 and 6 μg/mL of 11-dehydrosinulariolide treatment, respectively (Figure 1).

### 2.2. Apoptotic Assessment of A2058 Cells Treated with 11-Dehydrosinulariolide

**Figure 2:**
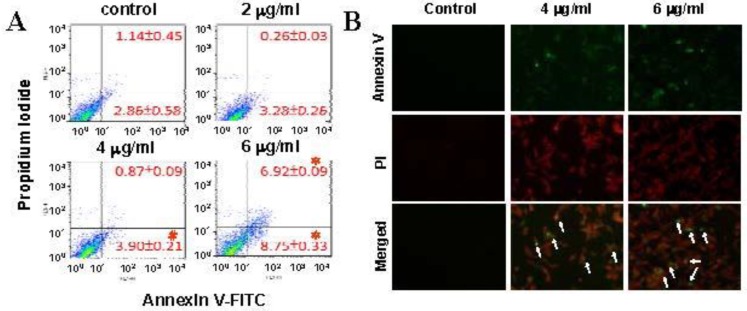
Flow cytometric data of 11-dehydrosinulariolide-induced apoptosis in A2058 melanoma cells. Detection of externalization of phosphatidylserine(PS) from cell membrane after 11-dehydrosinulariolide treatment stained by annexin V-FITC/PI analysis. (**A**) Early apoptotic cells were increased after exposure to 4 and 6 μg/mL 11-dehydrosinulariolide and 6 μg/mL 11-dehydrosinulariolide elevated late apoptosis in melanoma cells (# *P* <0.05, *P <0.01). (**B**) Melanoma cells after 4 and 6 μg/mL 11-dehydrosinulariolide treatment were stained by PI (red) as well as annexin-V (green) and then observed using a fluorescent microscope (Olympus IX71 CTS, Chinetek Scientific, China). The apoptotic melanoma cells with fluorescence in 4 μg/mL and 6 μg/mL 11-dehydrosinulariolide treated groups were clearly visualized in contrast with control.

To investigate the apoptosis-inducing effects of 11-dehydrosinulariolide, A2058 melanoma cells exposed to 11-dehydrosinulariolide were analyzed using annexin V-FITC & PI staining on a flow cytometer. The induction of apoptosis in melanoma cells was determined by a flow cytometer based-annexin V staining. Treatments with 11-dehydrosinulariolide at 4 and 6 μg/mL enhanced the percentages of early apoptotic melanoma cells. As late apoptotic cells were also increased after treatment with 6 μg/mL 11-dehydrosinulariolide, it indicates that 11-dehydrosinulariolide possesses early and late apoptosis-inducing capacity in melanoma cells (Figure 2).

### 2.3. 11-Dehydrosinulariolide Triggers Mitochondrial Membrane Damage and Activation of Caspase-Dependent Pathway

**Figure 3:**
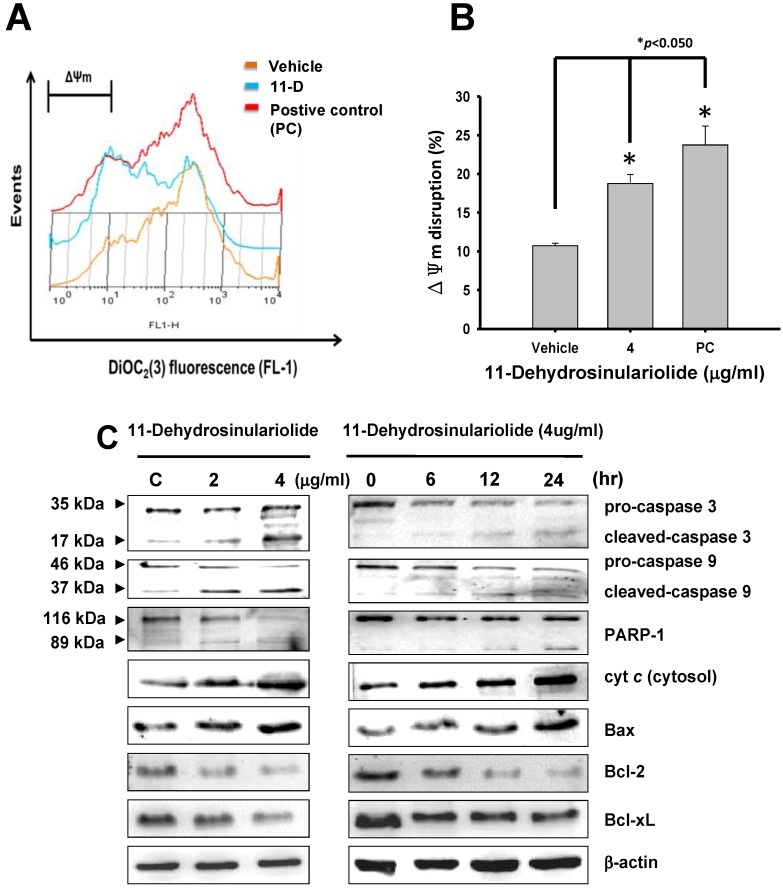
11-Dehydrosinulariolide induced apoptosis via mitochondria related pathway. (**A**) Measurement of mitochondrial membrane potential (ΔΨm) loss in A2058 melanoma cells after 4 μg/mL 11-dehydrosinulariolide treatment compared with vehicle and positive control 3-chlorophenylhydrazone (CCCP). (**B**) Loss of ΔΨm was significantly increased in the 4 μg/mL 11-dehydrosinulariolide treated group compared with vehicle (*P < 0.05), suggesting disruption of mitochondrial membrane. (**C**)Western blotting data showed the changes of cytosolic cytochrome *C*, pro-caspase-3, cleaved-caspase-3, pro-caspaes-9, cleaved-caspase-9, PARP-1, Bax, Bcl-2 and Bcl-xL expression in melanoma cells treated with 11-dehydrosinulariolide at different concentrations for 0–24 h. β-actin was used as the internal control.

Flow cytometric data displayed the loss of mitochondrial membrane potential induced by 11-dehydrosinulariolide, suggesting mitochondrial pathway is involved in 11-dehydrosinulariolide-induced apoptosis in melanoma cells (Figure 3A,B). To verify this mitochondrial dysregulation, we further analyzed several apoptotic markers including cytosolic cytochrome *C*, caspase-3, caspase-9 and PARP-1. Western blotting data showed dose- and time-dependent pro-caspase-9 and pro-caspase-3 down-regulation. Expression of caspase-3 (17 kDa proteolytic fragments) was elevated after 11-dehydrosinulariolide treatment. Similarly, cleaved caspase-9 fragment was observed at 37 kDa after the treatment. Enhancement of cytochrome *C* release from mitochondria into the cytoplasm was also found. A decrease of PARP-1 (116 kDa) as well as an increase of cleaved-PARP-1 (89 kDa) after 11-dehydrosinulariolide treatment was also shown by western blot analysis (Figure 3C).

### 2.4. 11-Dehydrosinulariolide Induces the Activation of ER Stress Pathway

**Figure 4:**
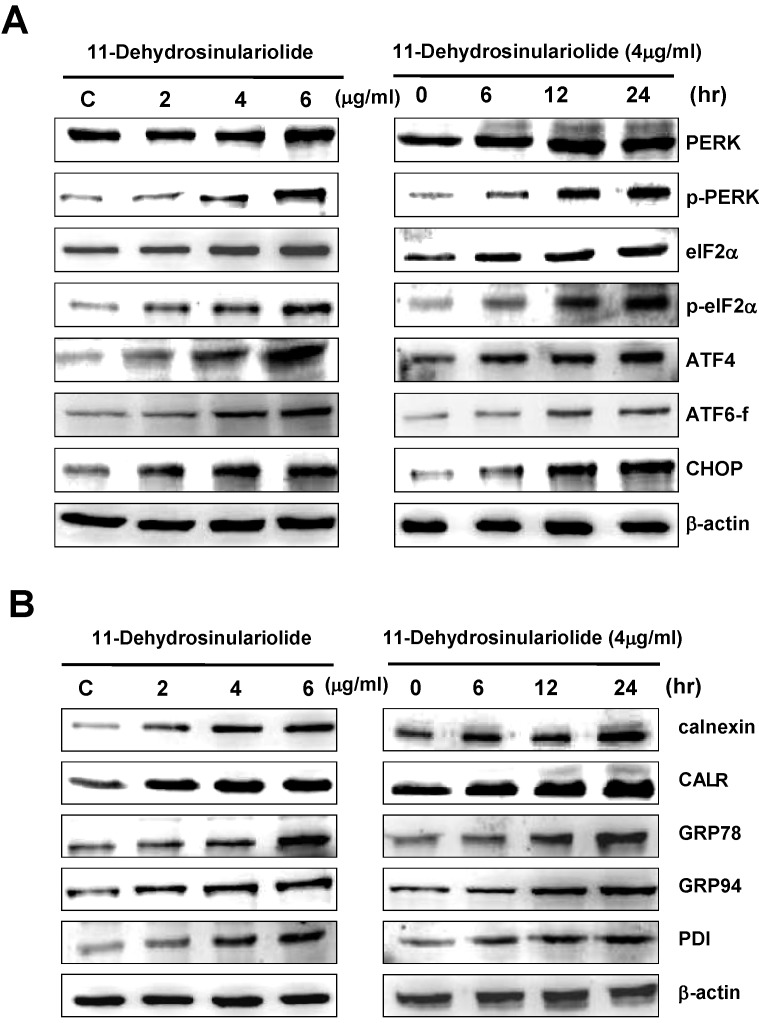
Exposure to 11-dehydrosinulariolide stimulated the factors of ER stress-mediated apoptotic pathway. (**A**) The changes of ER stress sensors PERK, p-PERK, eIF2α, p-eIF2α, ATF4, ATF6 fragment and CHOP were represented by immunoblotting; (**B**) Up-regulations of the ER stress related chaperones calnexin, CALR, GRP78, GRP94 and PDI are represented. β-actin was used as the internal control.

In the current study, regulations of three ER sensors, PERK, IRE1 and ATF6 as well as the ER chaperones, GRP78, GRP94, CALR, calnexin and PDI were verified by western blot. After 6–24 h exposure to 11-dehydrosinulariolide, p-PERK and p-eIF2α were increased, coupling with the unchanged normal forms. The transcription factor ATF4, a downstream signal of PERK-eIF2α pathway, was increased after 11-dehydrosinulariolide treatment in A2058 melanoma cells. Expression of CHOP, a hallmark of the ER stress-mediated apoptosis, was also increased (Figure 4A). In addition, the expression ER chaperones GRP78, GRP94, CALR, calnexin and PDI was dose- and time-dependently up-regulated after exposure to 11-dehydrosinulariolide in melanoma cells (Figure 4B).

**Figure 5:**
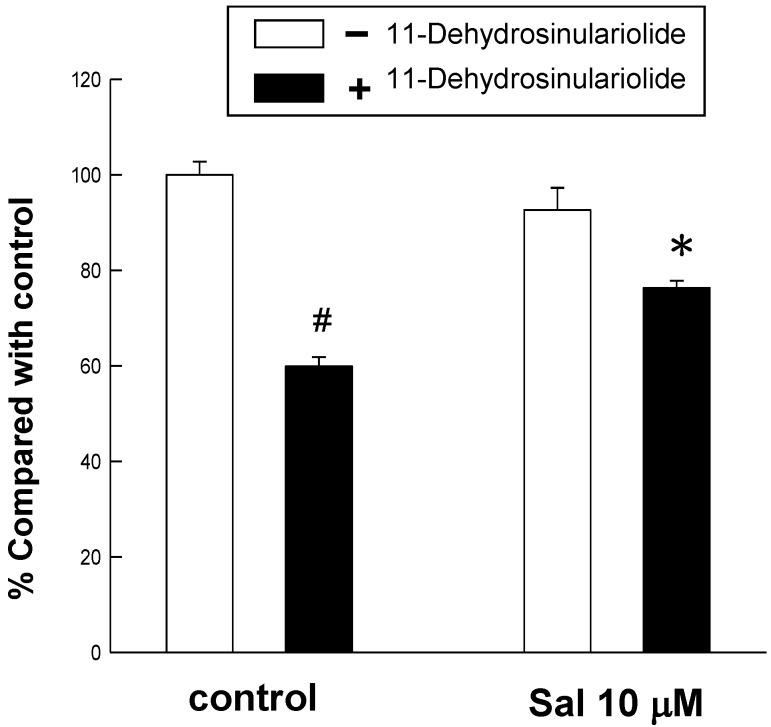
Partial restoration of 11-dehydrosinulariolide cytotoxic effects on A2058 melanoma cells by treatment with the apoptotic inhibitor salubrinal. MTT assay data showed the melanoma cell viability was significantly restored after the treatment with 10 μM salubrinal (#*P* < 0.05; *P < 0.01).

The ER stress inhibitory test was performed to further examine whether 11-dehydrosinulariolide-treated cells were rescued from apoptosis by pre-treating salubrinal, a selective ER stress inhibitor targeting eIF2α dephosphorylation. As a result, cell viability of the 11-dehydrosinulariolide-treated melanoma cells was significantly increased after pre-treatment with 10 μM salubrinal (Figure 5). This result supports the involvement of ER stress in the 11-dehydrosinulariolide-induced apoptosis.

## 3. Discussion

In this study, the cytotoxic effects and the potential mechanisms of apoptosis induced by 11-dehydrosinulariolide in melanoma cells were examined by MTT assay, cell migration assay, flow cytometry and immunoblotting. We have previously shown that the same compound from soft coral *Sinularia leptoclados* exerts anti-cancer activities against oral squamous cell carcinoma by anti-proliferative, anti-migratory and proteomic analysis [27]. Similarly, the results in this study indicated that 11-dehydrosinulariolide possesses anti-proliferative and anti-migratory effects against melanoma cells (Figure 1), suggesting broad anti-tumor activities exerted by the soft coral isolated compound 11-dehydrosinulariolide. In the current work, the flow cytometric results indicated that 11-dehydrosinulariolide induces both early and late apoptosis (Figure 2A,B). Apoptosis plays important roles in removing DNA-damaged cells from surrounding normal cells and aims to control the development of multicellular organisms and maintain tissue homeostasis [28]. The carcinogenesis and neoplastic progression of various human cancers are primarily due to deregulation or/and dysfunction of apoptotic signal pathways [29]. Therefore, novel apoptotic inducers indeed provide effective and promising therapies against human cancers. 

The mitochondria-dependent apoptotic pathway has been characterized by pro-apoptotic factors including the release of cytochrome *C*, activation of caspase-3/9 and Bax, as well as suppression of the anti-apoptotic proteins like Bcl-2 and Bcl-xL [18,30]. Our western blotting results found both time- and dose-dependent up-regulation of pro-apoptotic proteins such as cytosolic cytochrome *C*, cleaved-caspase-3, cleaved-caspase-9, and Bax while pro-caspase-3, pro-caspase-9 and anti-apoptotic factors Bcl-2 as well as Bcl-xL were down-regulated in 11-dehydrosinulariolide-treated melanoma cells (Figure 3C). These data suggested potential involvement of the caspase 3-, caspase 9- and mitochondria-dependent pathway in 11-dehydrosinulariolide-induced apoptosis in melanoma cells. Interestingly, these findings are quiet similar to the possible mechanisms of apoptosis induced by another soft coral extracted compound 13-acetoxysarcocrassolide in bladder cancer cells in our previous study, implicating some common cytotoxic and apoptosis-inducing characteristics exerted by the natural marine products against different tumor/cancer cells [23]. 

Additionally, evidence showed that the over-expression of pro-survival Bcl-2 or deficiency of pro-apoptotic Bax conferred protection against mitochondrial dysregulation [31,32]. The mitochondrial dysregulation is characterized by the loss of mitochondrial membrane potential and caspase-3/9 is subsequently activated [33]. In the current study mitochondrial membrane potential in melanoma cells was attenuated within 12 h under 11-dehydrosinulariolide treatment (Figure 3A,B). These results are rationally linked with the western blotting data of these mitochondrial apoptotic events such as enhancement of Bax expression, simultaneous inhibition of Bcl-2/Bcl-xL, subsequent Bax insert into mitochondria outer membrane, release cytochrome *C* and cleavage of caspase-3/9 in 11-dehydrosinulariolide-induced apoptosis in melanoma cells.

Another apoptotic hallmark protein, cleaved-PARP-1, also plays an important role in triggering apoptosis via a caspase-independent mechanism [34]. Investigation indicated that cleaved-PARP-1 initiates apoptosis-inducing factor (AIF)-mediated cell death through a mechanism requiring Bax and calpains but not cathepsins or caspases [35]. Our data showed that exposure to 11-dehydrosinulariolide results in cleavage of PARP-1 in A2058 melanoma cells in a time- and dose-dependent manner (Figure 3C). These results suggested that the cleaved-PARP-1 apoptotic event may be associated with mitochondria-mediated apoptosis through both caspase-dependent and caspase-independent pathways.

The apoptotic events after 11-dehydrosinulariolide treatment in melanoma cells were also found to be correlated with ER stress. The onset of ER stress is due to accumulation and aggregation of unfolded or misfolded proteins in the lumen of ER which initiates unfolded protein response (UPR) and ER-associated protein degradation (ERAD). The UPR and ERAD can restore normal ER function, whereas they are able to trigger apoptosis when the stress is prolonged or the adaptive responses fail [16,36]. The UPR is mediated via three ER transmembrane sensors, PERK, ATF6 and IRE1, which are capable of triggering self-rescuing or apoptotic responses through concerted and complex signaling pathways, respectively [37]. Transcriptional induction of ERAD components have been shown to depend on the IRE1-XBP1 pathway in mammals [38]. Therefore, ER stress sensors such as PERK and ATF6 were also analyzed to verify the involvement of ER stress in the 11-dehydrosinulariolide-induced apoptosis in this study. After 11-dehydrosinulariolide treatment, PERK and eIF2α were phosphorylated and resulted in elevation of p-PERK and p-eIF2α levels in a time- and dose-dependent manner. Transcription factor ATF4, a downstream signal of PERK-eIF2α pathway was also enhanced after 11-dehydrosinulariolide treatment (Figure 4). ATF4 can trigger a self-rescuing response by inducing the genes correlated with amino acid metabolism, redox reaction, stress response and protein secretion [39]. This factor was also shown to induce the pro-apoptotic transcription factor CHOP, which is an ER stress-mediated apoptotic executor [40].

The cleaved and activated form of another transcription factor ATF6, ATF6-f, was also increased under the same conditions of treatment (Figure 4A). Similarly, previous work by Yamamoto *et al.* (2007) found that activated ATF6 triggers a self-rescuing response via inducing ER chaperones (*i.e.*, GRP78, GRP94, PDI) and XBP1 [41] as ATF6 can also induce CHOP expression [42]. The up-regulation of CHOP has been revealed to cause growth arrest and apoptosis in typical ER stress events after tunicamycin and thapsigargin treatment [43,44]. Our immunoblotting results displayed that CHOP expression is up-regulated in the time-course and dose-dependent test (Figure 4). The ER stress inhibitor salubrinal suppresses dephosphorylation of p-eIF2α by targeting the specific phosphatase of eIF2α [20]. In the current study salubrinal partially abrogated 11-dehydrosinulariolide-induced cell death (Figure 5). Since more than one possible apoptotic pathway of 11-dehydrosinulariolide-induced cytotoxicity against melanoma cells was found in the current study, it is qute reasonable that melanoma cell viability recovery rate after salubrinal treatment is around 20%. Combining the data on ER stress-associated factors with the restorative result of ER stress-mediated apoptosis, it is highly indicated that 11-dehydrosinulariolide is able to induce ER stress-mediated apoptosis in melanoma cells, and the PERK/eIF2α/ATF4/CHOP as well as the ATF6/CHOP pathways are very likely to be involved in the apoptotic events. 

Our western blotting data also showed that up-regulations of ER chaperones (GRP78, GRP94, calnexin, CALR, and PDI) are found after 11-dehydrosinulariolide treatment in a dose- and time-dependent manner (Figure 4B). These ER chaperones have been indicated to be relevant to ER stress and the ER stress-mediated apoptotic factors shown above. GRP78 is a key indicator of ER stress responses and is able to control the activation of PERK, ATF6 and IRE1 through a binding-release mechanism [45] as GRP 94 is another indicator of ER stress (Little et al, 1994). Furthermore, investigation has revealed that the up-regulation of GRP78/GRP94 is mediated by single or combined actions of IRE1-XBP1 and ATF6 in ER stress responses [38,46,47]. Immunoblotting results of both ER-stress apoptotic mediators and ER chaperones GRP78/GRP94 were up-regulated in the current study, implying that 11-dehydrosinulariolide initiates ER stress responses and may activate the transcription of GRP78/GRP94 promoters. Other ER-resident chaperones such as calnexin, CALR and PDI constitute a chaperon system in ER, which has been shown to retain nascent unfolded *N*-linked glycoprotein and further processes them for subsequent folding and assembling [48]. It has been shown that elevated calnexin levels protect cells from apoptosis [49]. Meanwhile, calnexin over-expression has been found in ER stress-induced apoptosis in a fission yeast model [50] as this protein has also been found to regulate ER-stress-mediated apoptosis by a B-cell receptor-associated protein 31 (Bap31) binding in a dependent manner but independent of its chaperone function [51]. On the other hand, in the *Caenorhabditis elegans* model tunicamycin induced expression of CALR, which requires IRE1-dependent splicing of XBP1 mRNA but not ATF6 and PERK [52]. Investigation on the correlation between PDI expression and UPR has shown that the PDI level is regulated via activation of ATF6 and splicing of XBP1 mRNA [53,54]. Hence, it is proposed that the up-regulations of the ER chaperones calnexin, CALR and PDI after exposure to 11-dehydrosinulariolide are likely self-rescuing responses of the tumor cells or the onset of ER stress-mediated apoptosis induced by the treatment in the melanoma cells.

Taken together, in the current study we found that the natural marine compound 11-dehydrosinulariolide possesses anti-tumor and apoptosis-inducing effects on A2058 melanoma cells. Mitochondrial and ER stress-mediated pathways of the apoptosis were examined and partially revealed. The results in the current study suggest that 11-dehydrosinulariolide is an effective compound against formidable melanoma cells *in vitro*. The potentially dual apoptotic pathways were found in the cytotoxic activities exerted by 11-dehydrosinulariolide and the finding should be one of the essential clues for the development of melanoma therapies. Thus, the compound 11-dehydrosinulariolide is worthy of further investigation in animal model tests. Future studies on the cross-talk between ER stress- and mitochondria-mediated apoptosis induced by 11-dehydrosinulariolide in melanoma cells will be helpful to uncover its anti-tumor mechanism and also benefit the pharmaceutical development of anti-melanoma drugs. 

## 4. Materials and Methods

### 4.1. Materials

Rabbit anti-human ATF4, cleaved-ATF6 antibodies were obtained from ProteinTech Group (Chicago, IL, USA). Rabbit anti-human glucose-related protein 78 (GRP78), glucose-related protein 94 (GRP94), elF2-α, cytochrome *C* and poly ADP-ribose polymerase-1 (PARP-1) antibodies were from Epitomics (Burlingame, CA, USA). Antibodies against pro-caspase-3, cleaved caspase-3, pro-caspae-9, cleaved caspase-9, calnexin, calreticulin(CALR), protein disulfide isomerase (PDI), Bax, Bcl-xL and Bcl-2, PERK, phospho-PERK (p-PERK), phospho-elF2α (p-elF2α) and CHOP were from Cell Signaling Technology (Danvers, MA, USA). Rabbit anti-human β-actin antibodies were from Sigma. Goat anti-rabbit and horseradish peroxidase conjugated IgG was from Millipore (Bellerica, MA, USA). Cytochrome *C* releasing apoptosis assay kit was from Biovision (Mountain View, CA, USA). Cell Extraction Buffer was obtained from BioSource International (Camarillo, CA, USA). Protease inhibitor cocktail was from Sigma (St Louis, MO, USA). PVDF (polyvinylidene difluoride) membranes and Chemiliminescent HRP Substrate were acquired from Pierce (Rockford, IL, USA). 

### 4.2. Cell Culture and Treatments

A2058 melanoma cells were grown in DMEM with 4 mM L-glutamine adjusted to contain 1.5 g/L sodium bicarbonate and 4.5 g/L glucose, supplemented with 10% (v/v) FBS, 100 units/ml penicillin, 100 μg/ml streptomycin and 1 mM sodium pyruvate in a humidified atmosphere with 5% CO_2_ in air at 37 °C. When cells reached above 70% confluency, subculture was conducted at a split ratio of 1:6. A2058 cells were cultured in a 10 cm dish for each assay. 11-Dehydrosinulariolide (Figure 1) was isolated from the soft coral *Sinularia leptoclados*. Cells were added with different concentrations of 11-dehydrosinulariolide (2 μg/mL, 4 μg/mL, 6 μg/mL, and 8 μg/mL) and harvested after 24 h incubation. The ER stress inhibitor, salubrinal at 10 μM was used to pre-treat melanoma cells 1 h before 11-dehydrosinulariolide treatment. All the experiments were repeated three times to confirm reproducibility.

### 4.3. Cytotoxicity Assay

The cytotoxic effects of 11-dehydrosinulariolide against A2058 melanoma cells were assessed by MTT assay. A2058 cells were incubated in 96-well plates at 1 × 10^5^/well in the medium and 200 μL/well. After treatment with various concentrations of 11-dehydrosinulariolide for 24 h, each well was charged with 50 μLMTT solution (1 mg/mL in PBS) and the plates were incubated at 37 °C for 4 h. To achieve solubility of formazan crystals, 200 μL of DMSO was added to each well. The absorbance was measured at 595 nm on a microtiter plate ELISA reader while DMSO was used as blank. 

### 4.4. Cell Migration Assay

For cell migration assay, A2058 melanoma cells in serum-free media were seeded onto polycarbonate membranes (8.0 μm, BD Biosciences, CA, USA) in the culture inserts. Melanoma cells with or without 11-dehydrosinulariolide treatment were allowed to migrate for 24 h. After removing non-migrating cells on the upper site, migrated cells on the lower site were fixed with 100% methanol and stained with Giemsa (Merck, Germany), respectively. Migrated cells were observed and counted under optics at 100X magnification [55].

### 4.5. Assessment of Apoptosis and Mitochondrial Potential Changes by Flow Cytometry

The apoptosis induced by 11-dehydrosinulariolide in A2058 melanoma cells was determined using annexin V staining (Pharmingen, San Diego, CA) on FACScan a flow cytometer (Becton-Dickinson, Mansfield, MA, USA) [56]. A total of 1 × 10^6^ cells per 10 cm petri-dish was treated with control or 11-dehydrosinulariolide for 24 h and subsequently labeled with 10 μg/mL of annexin V-FITC. Apoptotic distribution of 11-dehydrosinulariolide-treated A2058 melanoma cells was measured by Cell-Quest software (Becton-Dickinson). The changes of Mitochondria membrane potential were measured by flow cytometry using cationic cyanine fluorescence dye DiOC_2_(3) (MitoProbe™, Invitrogen) according to the manufacturer’s instructions. Briefly, drug-treated cells were washed, resuspended with 1 mL PBS, and incubated with 50 nM of DiOC_2_(3) for 30 min at 37 °C in the dark. Cells were treated with a known mitochondrial uncoupling agent, 3-chlorophenylhydrazone (CCCP) as a positive control. Data were analyzed using the software FlowJo [57]. 

### 4.6. Protein Extraction and Estimation

A2058 cells were untreated or treated with different concentrations of 11-dehydrosinulariolide (0, 2, 4 and 6 μg/mL) for 24 h and then lysed with Cell Extraction Buffer (BioSource International, Camarillo, CA, USA) and protease inhibitor cocktail (Sigma).

### 4.7. Western Blot Analysis

After SDS-PAGE analysis of the treated samples and the controls under reducing conditions, the proteins on gel were transferred to a PVDF membrane (Millipore) for 1.5 h at 400 mA using Transphor TE 62 (Hoefer). Mitochondrial and cytosolic cytochrome *C* were separated using a cytochrome *C* releasing apoptosis assay kit (Biovision). The membranes were then incubated with human GRP78, GRP94, calnexin, CALR, PDI, cytosolic cytochrome *C*, caspase-3, cleaved-caspase-3, caspase-9, cleaved-caspase-9, Bax, Bcl-2, Bcl-xL, PERP, p-PERP, eIF2α, p-eIF2α, PARP-1, ATF4, cleaved-ATF6, CHOP and β-actin antibodies at 4 °C for 2 h or overnight. The membranes were washed three times in PBST (10 mM NaH_2_PO_4_, 130 mM NaCl, 0.05% Tween 20) and then probed with horseradish peroxidase conjugated antibodies (1:5000) for 1 h. After washing with PBST three times, the enzyme activities on the blot were visualized through chemiluminesence by adding ECL Western Blotting Reagents (Pierce).

### 4.8. Statistical Analysis

Data of cell migration assay, MTT assay, and flow cytometric analysis were pooled from three independent experiments and expressed as mean ± standard error of mean (SEM) and analysis of variance (ANOVA) followed by the Tukey-Kramer test performed on GraphPad InStat 3 (San Diego, CA, USA), to determine significant differences (*p* ≤ 0.05) between experimental groups [23].

## 5. Conclusion

In the present study, 11-dehydrosinulariolide induced apoptosis in A2058 cells was validated and further investigated. Further functional studies demonstrated that 11-dehydrosinulariolide induced dysregulation of mitochondria and ER stress pathway. This is the first report describing the effect of 11-dehydrosinulariolide on ER stress and mitochondrial dysfunction. Our results indicated that the involvement of 11-dehydrosinulariolide in inducing cytotoxicity in A2058 cells is through both PERK/eIF2α/ATF4/CHOP and ATF6/CHOP pathways, which suggests that 11-dehydrosinulariolide could be a potent anticancer drug for melanoma cancer treatment. 
